# The neural correlates of value hierarchies: a prospective typology based on personal value profiles of emerging adults

**DOI:** 10.3389/fpsyg.2023.1224911

**Published:** 2023-12-18

**Authors:** Jia-Qiong Xie, Yun Tian, Jia Hu, Ming-Ze Yin, Ya-Dong Sun, Yan-Jie Shan, Ke Chen, Gang Feng, Jiang Qiu

**Affiliations:** ^1^Faculty of Social Sciences, Chongqing University, Chongqing, China; ^2^Key Laboratory of Cognition and Personality (SWU), Ministry of Education, Chongqing, China; ^3^Sleep and NeuroImaging Center, Faculty of Psychology, Southwest University, Chongqing, China; ^4^Institute for Advanced Studies in Humanities and Social Sciences, Chongqing University, Chongqing, China; ^5^Faculty of Education, Southwest University, Chongqing, China; ^6^Office of Social Sciences, Chongqing University, Chongqing, China; ^7^School of Marxism, Beijing Normal University, Beijing, China; ^8^Faculty of Psychology, Southwest University, Chongqing, China; ^9^Southwest University Branch, Collaborative Innovation Center of Assessment Toward Basic Education Quality at Beijing Normal University, Beijing, China

**Keywords:** value hierarchies, interindividual differences, emerging adults, latent profile analysis, functional magnetic resonance imaging

## Abstract

**Introduction:**

Value hierarchies, as motivational goals anchored in the self-schema, may be correlated with spontaneous activity in the resting brain, especially those involving self-relevance. This study aims to investigate the neural correlates of value hierarchies from the perspective of typology.

**Methods:**

A total of 610 Chinese college students (30.31% women), aged 18 to 23, completed the personal values questionnaire and underwent resting-state functional magnetic resonance imaging.

**Results:**

The latent profile analysis revealed three personal value profiles: traditional social orientation, modernized orientation, and undifferentiated orientation. Neuroimaging results revealed that individuals with modernized orientation prioritized openness to change value, and this personal-focus is related to the higher low-frequency amplitude of the posterior insula; individuals with traditional social orientation prioritized self-transcendence and conservation values, and this social-focus is related to the stronger functional connectivity of the middle insula with the inferior temporal gyrus, temporal gyrus, posterior occipital cortex, and basal ganglia, as well as weaker functional connections within the right middle insula.

**Discussion:**

Taken together, these findings potentially indicate the intra-generational differentiation of contemporary Chinese emerging adults’ value hierarchies. At the neural level, these are correlated with brain activities involved in processing self- and other-relevance.

## Introduction

Understanding the personal values of youths is an extremely important issue, considering the prominent role of personal values in predicting a variety of psychological constructs in youth, such as personality traits (Fischer and Boer, 2015), identity (Lee et al., 2010), and behavior (McDonald et al., 2015). Personal value is defined by most researchers as a broad, ideal, and cross-situational goal (Rokeach, 1973; Schwartz, 1992). Individuals set the order of their values in terms of their relative importance, forming a hierarchy of values (Sagiv and Schwartz, 2022). Interindividual differences in value hierarchies are prevalent both within and across societies (Brosch et al., 2011; Sagiv and Schwartz, 2022), reflecting unique genetic heritage, personal experiences, social structure, and culture (Rokeach, 1973; Schwartz, 1992). Tradition, for example, could be highly valued in Eastern cultures but not in Western ones. For some, it could be the most important thing in their value hierarchy, but not for others.

**Figure 1:**
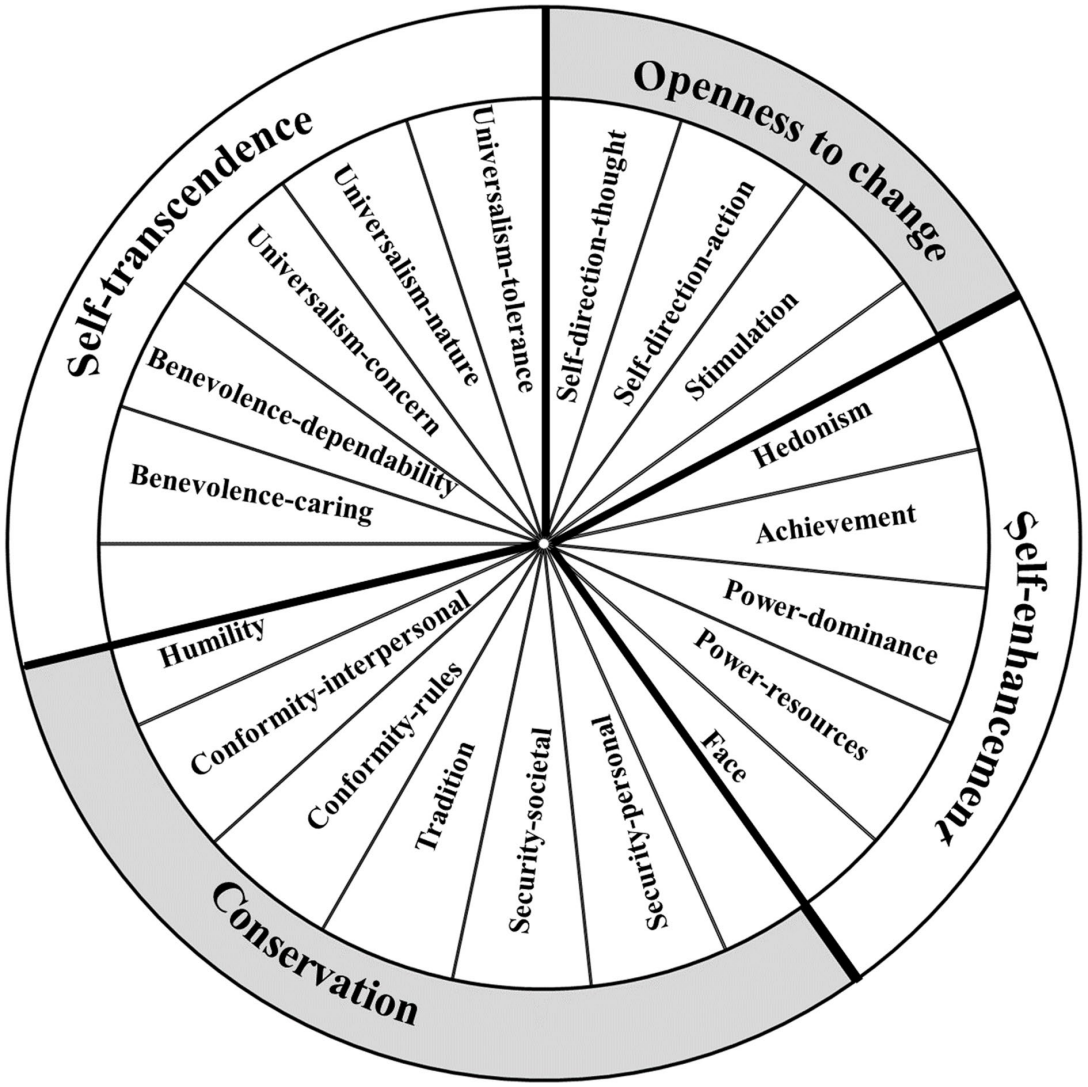
Circular motivational continuum of 19 values (Schwartz et al., 2012 [p. 669]).

The basic personal values theory is the theatrical framework of this study. Regarding personal value content, this theory has evolved from the original 10 basic personal values (Schwartz, 1992) to the refined 19 (Schwartz et al., 2012), see Figure 1. These values are likely to be universal since they were conceptually drawn from three universal requests of human existence: “needs of individuals as biological organisms, requisites of coordinated social interaction, and survival and welfare needs of groups” (Schwartz, 1992, p. 4). The structure of value hierarchy is another focus of the basic personal values theory. Schwartz et al. (2012) visualized social and psychological conflict or congruity between values in a circular structure, known as the circumplex model of values (see Figure 1). The array of values represents a motivational continuum, i.e., values placed next to each other have more compatible motivations, whereas those placed further apart are more conflicting. Empirically, Schwartz (1992) identified two motivational conflicts and theorized two pairs of higher-order value dimensions: openness to change (readiness for new ideas, actions, and experiences) versus conservation (self-restriction, order, and avoiding change), self-enhancement (pursuing one’s interests) versus self-transcendence (transcending one’s interests for the sake of others).

Social cognitive neuroscience provides a more intuitive analytical perspective in terms of revealing the neural correlations of personal values. Personal values are an important component of the self (Sagiv and Schwartz, 2022) and are easily associated with the self-related brain areas, with the dorsal/ventral/anteromedial medial prefrontal cortex (DMPFC/VMPFC/AMPFC), the anterior/posterior cingulate cortex (ACC/PCC), and the insula featuring most prominently. For instance, participants showed stronger blood oxygenation level-dependent (BOLD) signals in the VMPFC and DMPFC (Leszkowicz et al., 2021), as well as in MPFC and ACC (Teed et al., 2020) when rating self-transcendence values as opposed to self-enhancement values. Research on health behavior change found that self-transcendence values priming upregulated the activity of VMPFC, which predicted later decreases in sedentary behavior (Kang et al., 2018). Similarly, increased activation in VMPFC was observed when individuals with self-transcendence values were envisioning far-future events (Brosch et al., 2018). Additionally, Brosch et al. (2012) found that the distributed activation pattern in the insula and ACC allowed differentiating growth-type (self-transcendence and openness to change values) and protection-type values (self-enhancement and conservation values).

Qin et al. (2020) proposed that the neural model of self-processing involved three levels of interoceptive-processing, exteroceptive-processing, and mental-self-processing, among which the mental-self-processing involved MPFC and ACC and other extensive brain regions. A dorsal–ventral component of self- and other-reflection within the MPFC was observed in the literature; in particular, VMPFC are active for self-related (include close other) processing, while DMPFC are active for other- referential processing, more accurately for public other (for a review, see Denny et al., 2012; Murray et al., 2012). In this sense, previous findings imply that the concern for the interests of close people (benevolence value) and strangers (universal value) expressed by self-transcendence values are represented at the brain level by brain regions of self-related processing and public other-processing, respectively. The insula cortex, which is frequently co-activated with the ACC, is crucial for organizing behavior by combining external elements about stimuli with internal information about individual motivation, cognitive and self-awareness (Craig, 2009; Davey et al., 2016; Frewen et al., 2020). Interoceptive-, exteroceptive- and mental-self-processing all three levels involve insula activation, highlighting the basic role of internal-sensory signal integration in self-processing (Qin et al., 2020). Overall, the findings of task-based functional magnetic resonance imaging (FMRI) studies imply that as motivationally goals anchored in the self-schema, personal values are broadly associated with the brain regions of mental-self-processing (e.g., MPFC and ACC), and even interoceptive processing (e.g., insula) to exert its driving force for decisions and behaviors.

Presently, known personal value-related neuroscience studies are based primarily on variable-centered analyses wherein the biological indicators of personal values are revealed on the premise of sample homogeneity. Adding to this complexity, sample heterogeneity may contribute to the neural correlates of personal value in the overall sample, which may differ markedly from such correlations within some or all subgroups. Furthermore, the variable-centered approach is weak in identifying interindividual differences. Interindividual differences can easily be linked to taxonomic approaches. The person-centered method, as a worthy supplement to the traditional variable-centered perspective, focuses on organizing and integrating value hierarchies within individuals and abstractly systematizing individuals into an efficient categorization system of value hierarchies (Magun et al., 2016). Using a person-centered typological approach to identify heterogeneity within groups in terms of value hierarchies has yielded compelling results (e.g., Magun et al., 2016; Smack et al., 2017; Ungvary et al., 2018). Various statistical methods such as cluster analyses (Lee et al., 2011; Magun et al., 2016) and latent profile (class) analyses (LPA and PCA) (Moors and Vermunt, 2007; Magun et al., 2016; Ungvary et al., 2018) have been used to implement the typological approach in empirical studies, which identified two to five distinct heterogeneous groups with similar value hierarchy characteristics. For instance, researchers identified 5 subgroups in 29 European countries, namely “growth, weak person focus, strong person focus, weak social focus, and strong social focus” (Magun et al., 2016); 4 subgroups, namely anxiety-free, other-focused, self-focused, and undifferentiated in American and Israeli adolescents (Ungvary et al., 2018); and 2 subgroups, namely personal-focused and social-focused in Canadian college students (Smack et al., 2017). One explanation for the different numbers of subgroups is the different distribution of people in different countries (Magun et al., 2016; Iijima et al., 2020). These results highlight that the typological approach, as an appropriate strategy, can add valuable insights into taxonomy within and across countries.

In sum, despite existing value-related neuroscience research, the neural correlates of interindividual differences in value hierarchies have not been holistically considered. This study aims to characterize the individual difference in value hierarchies based on Schwartz’s theory and examines the potential neural correlates across these individual difference. Most empirical studies on the neural correlates of personal values mainly focus on populations in Western culture, where individualism flourishes. Some gaps, nevertheless, remain among those in Eastern culture that thrives on collectivism (Hofstede, 2001; Huang et al., 2019), especially among young people. In Chinese society, rapid economic development and urbanization in the past decades catalyzed the coexistence and integration of various values, with the characteristics of pluralistic construction, especially among the younger generation (Chen, 2015). Thus, Chinese emerging adults are a perfect fit for interindividual differences in value hierarchies owing to the prominent intragenerational and intergenerational differences in their value hierarchies. LPA was employed to characterize the different personal value profiles among Chinese emerging adults for achieving the first aim.

Schwarz’s personal values theory is based on universal human needs, and studies from approximately 100 nations support the near-universality of basic values content and circular order in this theory (Sagiv and Schwartz, 2022). This widespread cross-cultural acceptance may imply that values convey motives that have been evolutionarily conserved, reflected as innate individual differences in the brain (Zacharopoulos et al., 2016). Task-independent measures taken at rest can predict individual differences in brain level (Tavor et al., 2016). During rest, spontaneous brain dynamics maintain and optimize the brain’s generative models, providing top-down predictive signals for cognition and action in future interactions (Pezzulo et al., 2021). As broad goals across contexts, personal values are people’s guiding principles (Schwartz et al., 2012). Behavioral empirical research that reveals the effect of personal values on psychological constructs such as cognitive, emotional, and decision-making (for a review, see Sagiv and Schwartz, 2022) have validated this top-down guidance. If this is indeed the case, then individual differences in the value hierarchy may be reflected by differences in spontaneous fluctuations within and across brain regions during brain rest states. The former can be measured by the amplitude of low-frequency fluctuations (ALFF), a neuroimaging indicator of regional spontaneous neuronal activity (Zang et al., 2007; Gong et al., 2020); and the latter by the resting-state functional connectivity (FC), a neuroimaging indicator that captures the complex correlations in neural activity between brain regions, regardless of their spatial proximity (Power et al., 2011; Schaefer et al., 2018). This two robust dynamics indicators are sensitive to psychological differences in the neuropsychological literature (e.g., Wei et al., 2014; Simon et al., 2020). Thus, resting-state fMRI was employed, as well as ALFF and resting-state FC were analyzed to examine the neural correlates of different personal value profiles for achieving the second goal of this study.

Personal values are the core component of the self (Sagiv and Schwartz, 2022). Value hierarchies are critically involved in the organization of one’s self-schema (Brosch et al., 2012; Zacharopoulos et al., 2016). This theoretical anticipation, along with converging aforementioned variable-centered results regarding the neural correlates of personal values led us to expect that the spontaneous fluctuations within and across brain regions related to the self-schema, such as prefrontal cortex, cingulate cortex, and insular cortex may allow differentiating personal value profiles. Thus, the prefrontal cortex, cingulate cortex, and insular cortex were chosen as 3 regions of interest (ROIs) to enhance the power of the study.

## Methods

### Participants

The main data comprises individuals in our ongoing project, the Neuroimaging Multi-omics Project of College Students Personality Traits. For this study, participant exclusion criteria were (a) a history of neurological or psychiatric disorders and (b) contraindications to MRI scans. 617 right-handed undergraduate students aged between 18 and 23 years (188 men, mean age = 19.6 ± 2.08 SD) from Southwest China were recruited. Seven subjects dropped out of the FMRI scan, and finally the questionnaire data and brain imaging data of 610 participants were obtained and analyzed. Because this is the first study to directly investigate the neural correlation of value hierarchy differences, it was difficult to estimate the necessary sample size *a priori* based on existing literature. Instead, we collected data from a reasonably large sample and computed post-hoc sensitivity using G*Power (Faul et al., 2009). With an *α* of 0.05, a *β* of 0.80 (as recommended by Field, 2013), and 3 groups, we find that our tests are sensitive to *f*^2^ value of 0.13. Thus, 610 subjects were an adequate sample size. Written informed consent was obtained from all participants, and they were paid for their time.

### Personal values assessment and analysis

#### Personal value assessment

The Personal Values Questionnaire (PVQ) by Schwartz et al. (2012) was used to assess personal values using 48 items related to 19 basic personal values. These values were grouped into two bipolar value dimensions, including openness to change vs. conservation, and self-transcendence vs. self-enhancement. Each of the 48 items offered verbal portraits of different people, and the respondents were asked to evaluate how similar they were to the portrait using a 6-point Likert scale (1 = very much like me to 6 = not like me at all). Sample items assessing two bipolar value dimensions include statements such as “being creative is important to him/her” (openness to change), “it is important to him/her to maintain traditional values or beliefs” (conservation), “he/she wants everyone to be treated justly, even people he/she does not know” (self-transcendence), and “being wealthy is important to him/her” (self-enhancement). According to Schwartz et al. (2012), we interpret these degrees of subjective similarity as indicators of the importance of the corresponding values. In this sample, the Cronbach’s alpha coefficients of two bipolar value dimensions ranged from 0.86 for self-transcendence to 0.91 for conservation, showing high reliability.

#### Statistical analysis of personal value

First, considering that the individual bias of the participants (for example, some respondents’ scoring range is basically only 4–6 or only 1–3) may lead to the deviation in the ranking of value importance, we converted the absolute importance score of each value item to the relative importance score as suggested by Schwartz (2005) to control this deviation. Specifically, we calculated the average score across the 48 items and subtracted it from each of the 48 original raw scores, before calculating the scores for the 19 value types and two bipolar value dimensions.

Second, based on the literature of interindividual variations in value hierarchy (e.g., Ungvary et al., 2018), LPA was used to identify the optimal number of latent profiles to characterize a sample of Chinese emerging adults’ scores in two bipolar value dimensions. We used maximum likelihood estimation with robust standard errors in Mplus 8.3. The fit indices, parsimony, distinctiveness, interpretability, and profile size were considered to choose the optimal profile solution. Lower Akaike information criterion (AIC), Bayesian information criterion (BIC), and sample-size-adjusted Bayesian information criterion (aBIC) values indicated an optimal model fit to the data (Nylund et al., 2007). Significant *p*-values (*p* < 0.05) of the adjusted Lo-Mendell-Rubin (LMR) test or Bootstrapped Likelihood Ratio Test (BLRT) indicated that the model with *k* profiles is a better fit for the data than a model with *k* − 1 profile (Lo et al., 2001). A higher entropy value indicates that the profile solution has better goodness-of-fit, with values equal to or greater than 0.80, demonstrating a more accurate classification (Clark, 2010). We estimated the random sets of starting values with 2,000 seeds and 500 iterations to avoid local maxima. After the profiles were determined, Wilcoxon signed-rank test was used to analyze the difference in the relative scores of the two pairs of value dimensions in each profile.

### Imaging data acquisition and analysis

#### Imaging data acquisition and preprocessing

Images were acquired using a 3 T Prisma scanner (Siemens Medical Systems, Erlangen, Germany). During scanning, participants were in a supine position and were also required to keep their eyes open and look at the white cross over the screen without thinking about anything specific. Additionally, to further reduce head motion, circularly polarized head coil and foam padding were jointly used. 240 volumes were acquired using a gradient Echo Planar Imaging (EPI) sequence. Scanning parameter are provided in detail in Supplementary materials.

Data Processing and Analysis for Resting-State fMRI Advanced Edition (DPARSF A) on Statistical Parametric Mapping (SPM, Version 8.0) (http://rfmri.org/dpabi; Yan et al., 2016) was used to analyze the resting-state fMRI data. 240 volumes were preprocessed by slice-timing, motion-correcting, spatial normalizing, and spatial smoothing (see Supplementary materials). Participants with maximal head motion greater than 2 mm translation or 2.0° rotation were excluded from the study. The preprocessed data were parcellated using the Dosenbach Atlas, which includes 142 cortical regions and 18 subcortical regions (Dosenbach et al., 2010). The atlas defines 10 insula regions, 19 prefrontal cortex regions, and 4 cingulate cortex regions.

#### Imaging data analysis

The ALFF was used to calculate the intensity of spontaneous brain activity in the resting state. First, the preprocessed image time series was transferred to the frequency domain using a fast Fourier transform to obtain the power spectrum (Zang et al., 2007). Second, the square root of each frequency in the power spectrum was calculated. Thereafter, the mean value of each voxel was calculated within the frequency range of 0.008–0.09 Hz to obtain the ALFF, and then converted it to normal distribution with Fisher’s z transformation (zALFF). ROIs with profile differences in zALFF was used as seed for FC analysis. The Pearson correlation between the time courses of seed and the time courses of other cortical brain regions was calculated separately to construct seed-based whole-brain FC networks using the Functional Connectivity Toolbox (http://www.nitrc.org/projects/conn; Whitfield-Gabrieli and Nieto-Castanon, 2012). Fisher’s R-Z transformation was then used to transform the correlation coefficient matrix into *z* matrix to improve normality (zFC).

One-way analysis of variance (ANOVA) was employed to investigate the zALFF and zFC differences among value profiles. Additionally, the significant results tested in one-way ANOVA on zALFF and zFC were used to further calculate the Pearson correlation with the two bipolar value dimensions. Statistical analyses were performed using MATLAB version R2017b (The MathWorks Inc., Natick, MA) and SPSS version 25 (SPSS Inc., Chicago, IL). Testing was two-tailed with an *α*-level of 0.05, which was false discovery rate (FDR) correction for one-way ANOVA and Bonferroni correction for multiple comparisons and Pearson correlation analysis (*p* < 0.05).

Epsilon-insensitive support vector classification (SVC) was used to validate the neural correlates of personal value profiles. Firstly, zALFF and zFC with significant differences between personal value profiles were selected for feature training, while those without significant differences were removed from the training features. Subsequently, the Gaussian radial basis function (RBF) of kernel was used to perform model training. We applied Stratified 10-fold cross-validation (10F-CV) to determine the optimal hyper-parameter. Finally, the area under the receiver operating characteristic curve (AUC) and classification accuracy were used to assess the classification performance. We used Stratified 10F-CV and averaged the classification results to generate the overall accuracy. We performed permutation tests 1,000 times (*p*_pt_ < 0.05) to ensure that the classification results were significantly better than random. This pipeline was implemented by using Scikit Learn v1.1.2 on PyCharm (Pedregosa et al., 2011).

## Results

### Latent profile models of personal values

Models with one to five potential profiles were established. Table 1 presents the fit indices of the models. Evidently, as the number of categories increases, the values of AIC, BIC, and aBIC decrease. However, the non-significant LMR of the four-profile model (*p* = 0.24) indicated that the model did not significantly improve as the number of categories increased from three to four, and the three-profile model was preferable to the four-profile model. Additionally, the entropy value of the 3-profile model was 0.82, indicating high classification accuracy. Therefore, the three-profile model was determined to be optimal. Given the gender asymmetry evident in our sample, cross tabulation analysis was employed to further examine the effect of gender on profile creation, and the results showed no significant gender differences among the three potential profiles, *χ*^2^ = 3.88, *p* = 0.14.

**Table 1:** Fit statistics for the latent profile analysis.

Profile	AIC	BIC	aBIC	Entropy	LMR	BLRT
1	12882.03	12972.80	12921.96	1		
2	2480.96	2538.33	2497.06	0.58	0.01	0.00
3	2332.49	2411.93	2354.78	0.82	0.00	0.00
4	2221.55	2323.06	2250.04	0.76	0.24	0.00
5	2170.46	2294.04	2205.14	0.78	0.37	0.00

**Figure 2:**
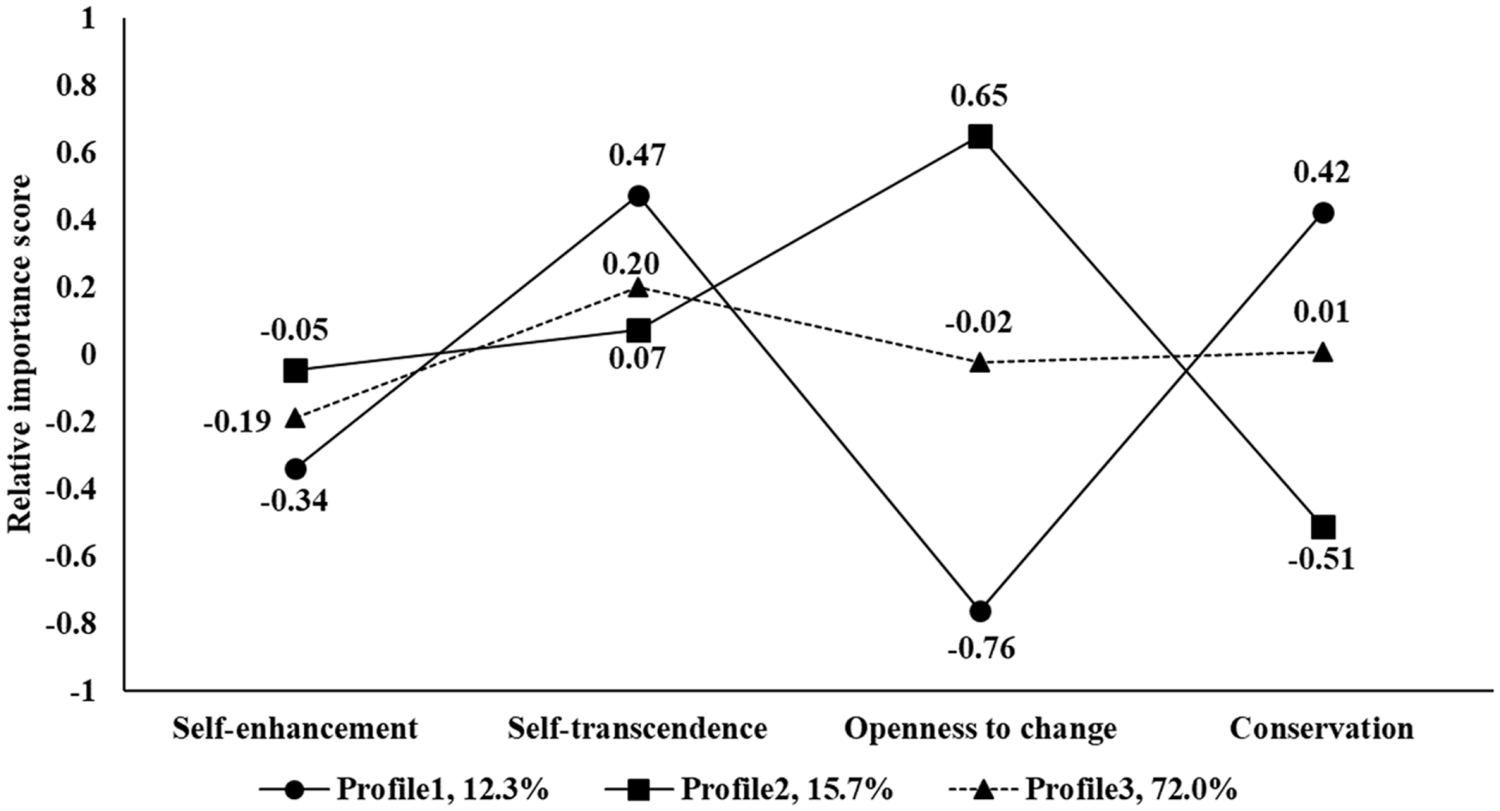
Latent profiles of personal values. The *Y*-axis value is the relative importance score. The larger the value, the more important the value dimension in the individual’s value hierarchy.

Figure 2 displays the latent profile sizes and relative importance score for the three-profile model of personal values. There were significant differences among the three profiles in the priority dimensions of in the priorities of self-enhancement values (*F*_(2, 607)_ = 8.46, *p*_corr_ < 0.001, *η*^2^*_p_* = 0.03, profile 1 < profile 3 < profile 2), self-transcendence values (*F*_(2, 607)_ = 32.51, *p*_corr_ < 0.001, *η*^2^*_p_* = 0.10, profile 1 > profile 3 > profile 2), openness to change values (*F*_(2, 607)_ = 415.12, *p*_corr_ < 0.001, *η*^2^*_p_* = 0.58, profile 1 > profile 3 > profile 2), and conservation values (*F*_(2, 607)_ = 468.46, *p*_corr_ < 0.001, *η*^2^*_p_* = 0.61, profile 1 < profile 3 < profile 2).

Profile 1 accounted for 12.3% of the whole sample, in which individuals’ priority of self-transcendence values is higher than that of self-enhancement values (0.47 vs. −0.34, *Z* = −6.30, *p* < 0.001), and the priority of conservation values is higher than that of openness to change values (−0.76 vs. 0.42, *Z* = −7.12, *p* < 0.001). These results suggested that they are socially focused, caring about the consequences for other people or for established institutions. This profile was labelled as *traditional social orientation* based on the basic personal values theory and the Chinese college students’ value hierarchy categorization results by Xie et al. (2022).

For the value hierarchy of the individual in Profile 2 (15.7% of the entire sample), no significant difference was observed in the priority of self-transcendence values and self-enhancement values (*Z* = −0.69, *p* = 0.49), while the priority of openness to change values is higher than that of conservation values (0.65 vs. −0.51, *Z* = −8.10, *p* < 0.001). These results revealed a shift in value orientation in modern Chinese society, as proposed by Zeng and Greenfield (2015), from the emphasis on loyalty to authority to independent thought, action, and feeling, as well as an openness to challenge and change. Thus, profile 2 is labeled as a *modernized orientation*.

Profile 3 was the largest class, comprising 72% of the entire sample. For the value hierarchy of individual in this profiles, no difference in priority was observed between the self-transcendence values and self-enhancement values (*Z* = −2.33, *p* = 0.07), nor between the openness to change values and conservation values (*Z* = −1.49, *p* = 0.14). According to these results, those in profile 3 do not seem to have a distinct value priority – a concept known as undifferentiated in earlier literature (Ungvary et al., 2018; Grapsas et al., 2023). Using this name, the *undifferentiated orientation* of profile 3 is identified.

### zALFF differences among personal value profiles

**Figure 3:**
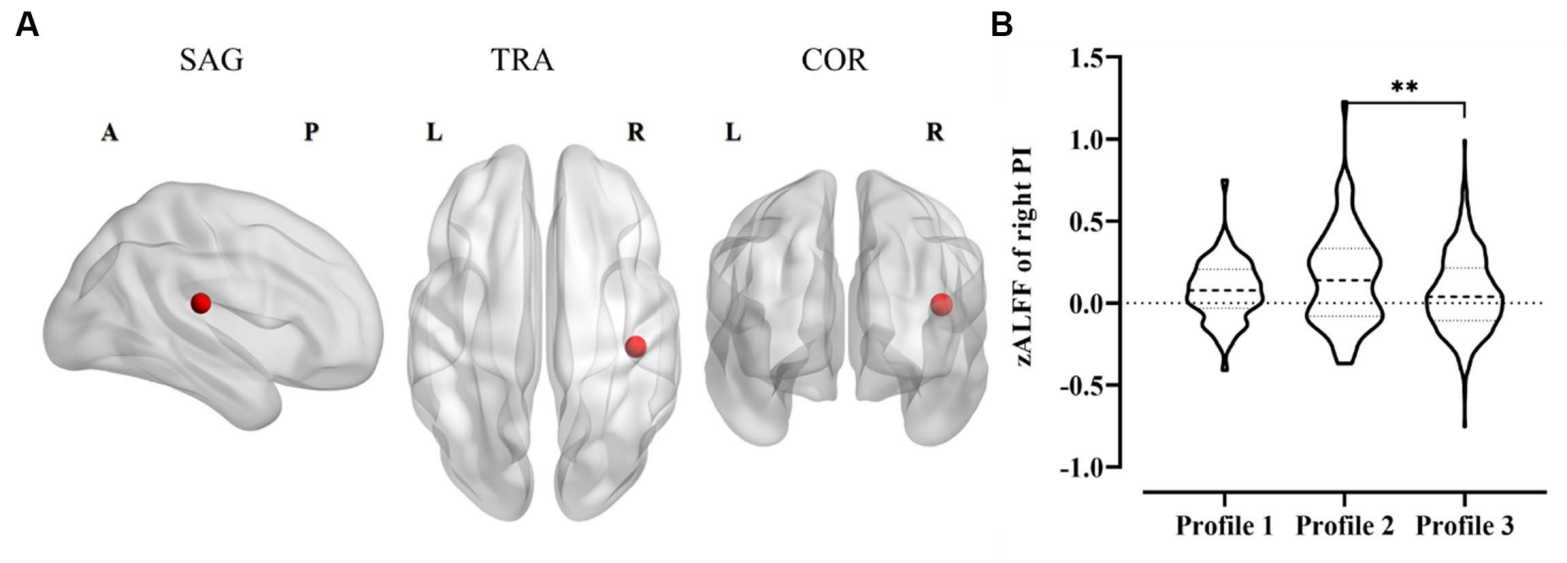
zALFF differences among personal value profiles. **(A)** zALFF difference in the right posterior insula (Montreal Neurological Institute (MNI) coordinates of red dot: *x* = 42, *y* = 24, *z* = 17) among personal value profiles. SAG, sagittal view; COR, coronal view; TRA, transversal view; A, anterior; P, posterior; L, left hemisphere; R, right hemisphere. **(B)** Multiple comparisons of zALFF differences in the right posterior insula among three personal value profiles. Violin plots show means (thick dotted line), standard deviation (fine dotted line), and distributions of parameter values. PI, posterior insula, ^**^p_corr_ < 0.01.

Figure 3 shows the zALFF differences among the three personal value profiles. One-way ANOVA test using FDR correction showed that there were spontaneous activity differences in the right posterior insula (PI) across the different personal value profiles, *F*_(2, 607)_ = 5.41, *p*_corr_ < 0.05, *η*^2^*_p_* = 0.02. However, no significant difference was found between personal value profiles for zALFF of other ROIs (see the Supplementary Table S3). The Bonferroni correction was applied with a factor of 10 (10 insular subregions) to account for multiple comparisons (*p*_corr_ = 10 × *p*_uncorr_). Multiple comparisons showed that profile 2 participants had higher spontaneous activity in the right PI than those in profile 3, *p*_corr_ < 0.01, 95% CI [0.03, 0.16]. However, spontaneous activity differences in the right PI between profile 1 and profile 2, or between profile 1 and profile 3 were not observed.

**Figure 4:**
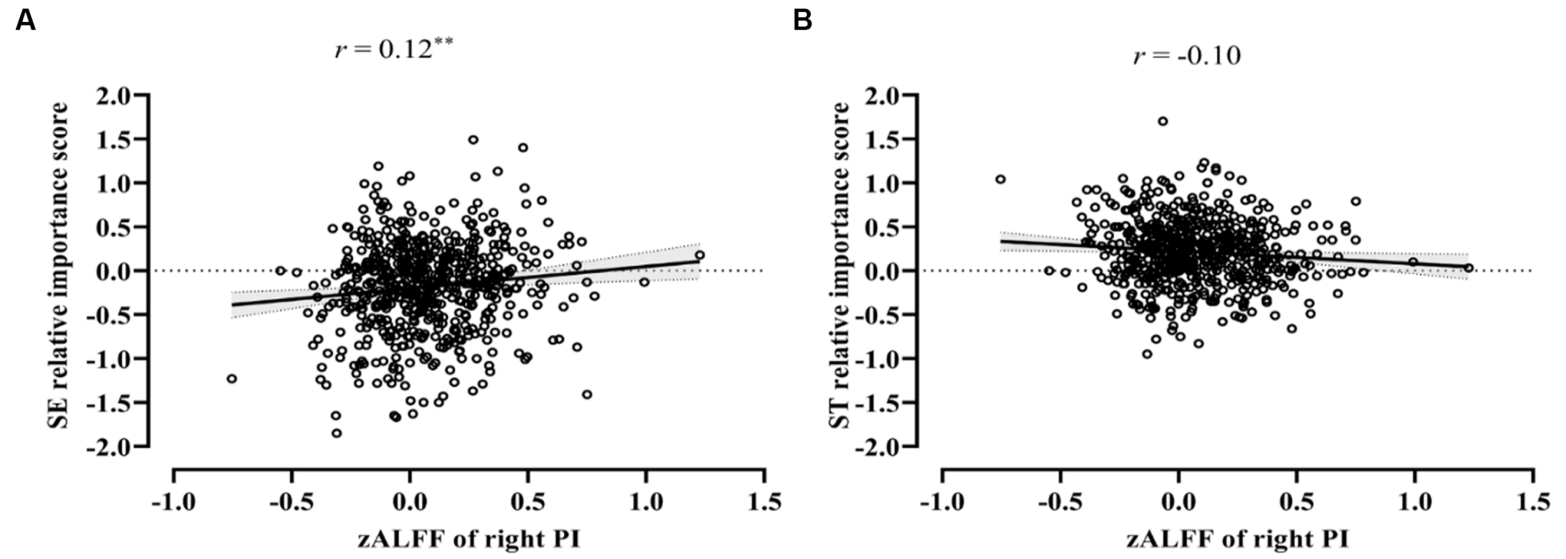
Correlation of value dimensions and zAFLL in the right posterior insula. **(A)** The significant positive correlation between self-enhancement values and zALFF in the right posterior insula. **(B)** The insignificant correlation between self-transcendence values and zALFF in the right posterior insula. ST, self-transcendence; SE, self-enhancement; PI, posterior insula. The solid black line is the best-fitting regression line, Error band represents 95% CI, ^**^p_corr_ < 0.01.

Subsequently, the correlation analysis between the two bipolar value dimensions and zALFF of the right PI was conducted. After Bonferroni correction with a factor of four (4 higher-order value dimensions, *p*_corr_ = 4 × *p*_uncorr_) was performed, the results indicated that the spontaneous activity of the right PI was significantly correlated negatively with self-enhancement (*r* = 0.12, *p*_corr_ < 0.05, 95% CI [0.04, 0.19], Figure 4A), while not with self-transcendence (*r* = −0.10, *p*_corr_ = 0.07, 95% CI [−0.17, −0.02], Figure 4B).

### zFC differences among personal value profiles

**Figure 5:**
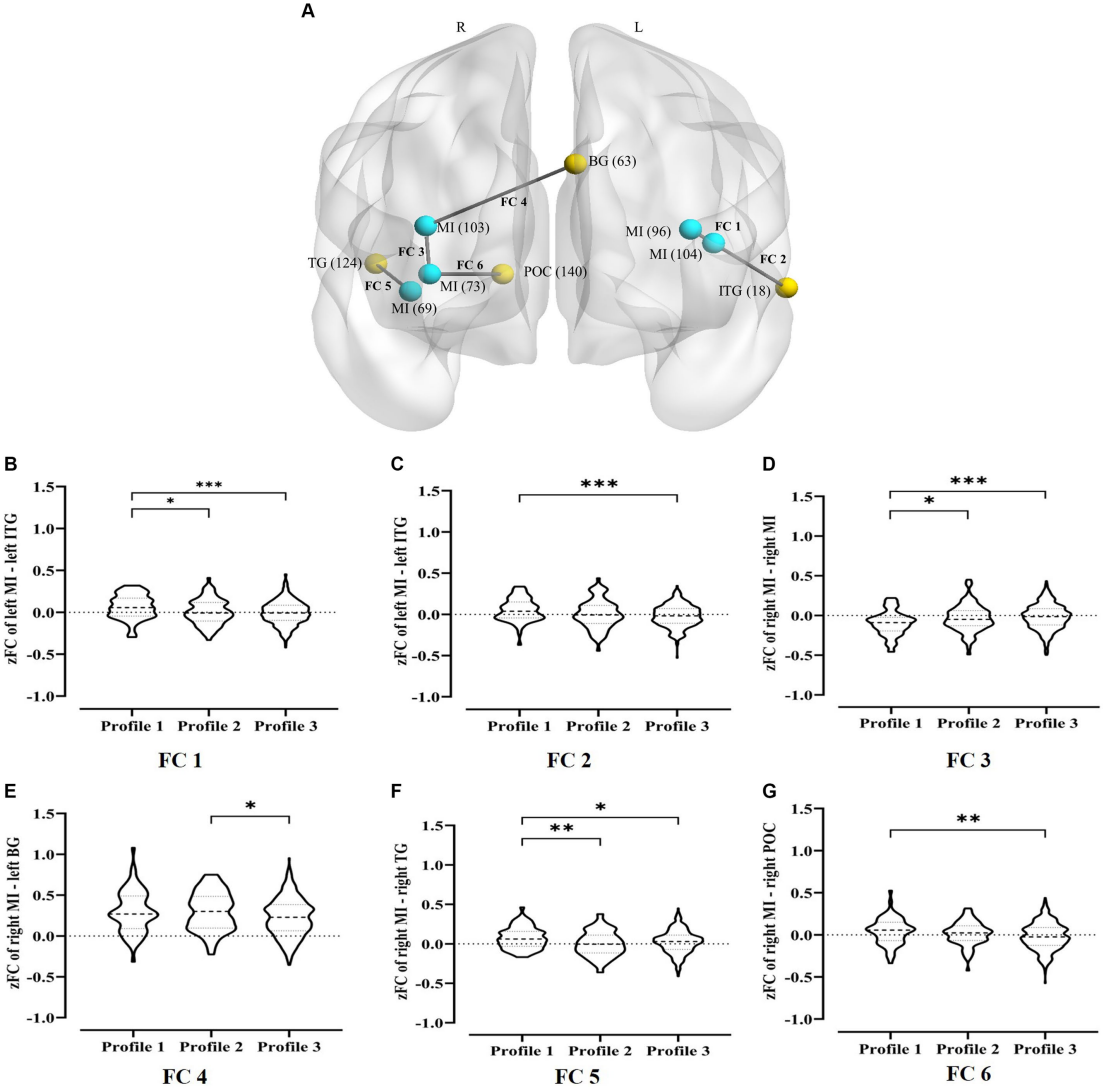
FC intensity differences between personal value profiles. **(A)** Six insula-based FC with significant intensity differences among three personal value profiles. Labels for cortical regions defined in the Dosenbach Atlas template are in parentheses; the blue dots are ROI 1 and the yellow dots are ROI 2; L, left hemisphere; R, right hemisphere. **(B–G)** Multiple comparisons of FC intensity between the left middle insula and left inferior temporal gyrus **(B,C)**, within the right middle insula **(D)**, between the right middle insula and left basal ganglia **(E)**, between the right middle insula and right temporal gyrus **(F)**, and between the right middle insula and right posterior occipital cortex **(G)** among three personal value profiles. MI, middle insula; ITG, inferior temporal gyrus; BG, basal ganglia; TG, temporal gyrus; POG, posterior occipital cortex. ^*^p_corr_ < 0.05, ^**^p_corr_ < 0.01, and ^***^p_corr_ < 0.001. More details of these FC intensities are described in Table 2.

Insula regions with profile differences in zALFF was used as seed for FC analysis. One-way ANOVA was performed to investigate the personal value profile differences of insula-based FC intensity. Figure 5 and Table 2 present the results. We conducted FDR correction on the one-way ANOVA results for zFC (*p* < 0.05). *Post hoc* multiple comparisons using Bonferroni correction revealed that profile 1 participants had higher FC intensities in the left middle insula (MI) with left inferior temporal gyrus (ITG), right MI with right temporal gyrus (TG), and right MI with right posterior occipital cortex (POC) than profile 3 participants; they also had higher FC intensities in the left MI with left ITG, and right MI with right TG than profile 2 participants; but lower FC intensity within the right MI than profile 2 and 3 participants. Additionally, profile 2 participants had higher FC intensity of the right MI with left basal ganglia (BG) than profile 3 participants.

**Table 2:** Comparison of six insula-based FC intensities among three personal value profiles.

FC	ROI 1			ROI 2			ANOVA			Multiple comparisons		
	Labels	Regions	MNI-coordinates (*x*, *y*, *z*)	Labels	Regions	MNI-coordinates (*x*, *y*, *z*)	*F*_(2, 607)_	*p*_uncorr_	*p*_corr_	*η* ^2^ *_p_*	Comparison	*p*_corr_
FC 1	96	Left MI	−42, −3, 11	18	Left ITG	−61, −41, −2	6.95	0.00	0.01	0.02	P1 > P2	0.03
											P1 > P3	0.00
FC 2	104	Left MI	−36, −12, 15	18	Left ITG	−61, −41, −2	8.67	0.00	0.00	0.03	P1 > P3	0.00
FC 3	103	Right MI	33, −12, 16	73	Right MI	32, −12, 2	7.72	0.00	0.01	0.03	P1 < P2	0.04
											P1 < P3	0.00
FC 4	103	Right MI	33, −12, 16	63	Left BG	−6, 17, 34	5.65	0.00	0.04	0.02	P2 > P3	0.02
FC 5	69	Right MI	37, −2, −3	124	Right TG	46, −62, 5	5.46	0.01	0.04	0.02	P1 > P2	0.00
											P1 > P3	0.03
FC 6	73	Right MI	32, −12, 2	140	Right POC	13, −91 2	5.91	0.00	0.03	0.02	P1 > P3	0.04

The *p*_corr_ value in the ANOVA results refers to that after FDR correction; the *p*_corr_ values in the multiple comparisons refers to those after Bonferroni correction. *η*^2^*_p_*, partial eta squared; P1, profile 1 (traditional social orientation); P2, profile 2 (modernized orientation); P3, profile 3 (undifferentiated orientation); MI, middle insula; ITG, inferior temporal gyrus; BG, basal ganglia; TG, temporal gyrus; POG, posterior occipital cortex.

**Figure 6:**
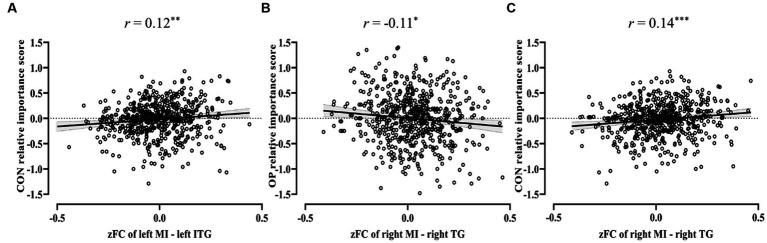
Correlation of value dimensions and insula-based FC intensity. **(A)** The significant positive correlation between conservation values and the FC of left middle insula – left inferior temporal gyrus. **(B)** The significant negative correlation between openness to change values and the FC of right middle insula – right temporal gyrus. **(C)** The significant positive correlation between conservation values and the FC of right middle insula – right temporal gyrus. MI, middle insula; ITG, inferior temporal gyrus; TG, temporal gyrus. The solid black line is the best-fitting regression line. Error band represents 95% CI. ^*^p_corr_ < 0.05, ^**^p_corr_ < 0.01, and ^***^p_corr_ < 0.001.

The correlation analysis between the two bipolar value dimensions and six insula-based FC intensities was performed. The results corrected by Bonferroni correction with four factors (4 higher-order value dimensions, *p*_corr_ = 4 × *p*_uncorr_) indicated that the FC intensity between the left MI and left ITG was significantly correlated positively with conservation (*r* = 0.12, *p*_corr_ = 0.01, 95% CI [0.04, 0.20], Figure 6A); the FC intensity between the right MI and the right TG was significantly correlated negatively with openness to change (*r* = −0.11, *p*_corr_ = 0.04, 95% CI [−0.18, −0.03], Figure 6B), while it was significantly correlated positively with conservation (*r* = 0.14, *p*_corr_ = 0.00, 95% CI [0.06, 0.22], Figure 6C).

The SVC achieved a significant classification performance in distinguishing three personal value profiles by ALFF in the right PI, FC between the MI and ITG/TG/BG/POC, and FC within the MI (AUC = 0.63–0.72, *p*_pt_ < 0.001, Accuracy rate = 62%–72%, *p*_pt_ < 0.05). For further details on the classification performance evaluation, see the Supplementary Table S4.

## Discussion

From a typology perspective, the present study characterized the personal value profiles of Chinese emerging adults using LPA. The LPA results revealed three heterogeneous personal value profiles, supporting the pluralistic construction and intragenerational differentiation of contemporary Chinese emerging adults’ value hierarchy. Specifically, a value profile (profile 1) consistent with Chinese cultural values was found and labeled as *traditional social orientation*. In this profile, self-transcendence and conservation were highly prioritized, while self-enhancement and openness to change were at the bottom of the value hierarchy. This reflects the collectivistic value in traditional Chinese culture, that is, emphasis on harmonious relationships and hierarchical order, valuing stability over freedom, groups and family over individuals, encouraging undemonstrative and unassuming behavior (King and Bond, 1985; Cai et al., 2010; Zeng and Greenfield, 2015). Individuals in profile 2 prioritized openness to change, and thus, this profile was labeled as *modernized orientation*. This is consistent with the research findings of Zeng and Greenfield (2015) wherein individualist values were continuously enhanced by analyzing the frequency of individualist words used by the Chinese people. According to the theory of social change and human development by Greenfield (2009), urbanization and modernization may result in the alteration of cultural values in an individualistic direction. Indeed, Chinese society has become increasingly market-oriented and modernized, and modern oriented values have gradually been accepted by people, especially the younger generations (Chen and Chen, 2010; Zeng and Greenfield, 2015). Schools and parents also began to encourage students to develop independence, self-expression, and self-exploration, which were ignored in traditional Chinese culture (Way et al., 2013).

Critically, the third and largest profile (72%) that emerged was labeled as *undifferentiated orientation* because of its moderate endorsement of all four values. This result contradicted Schwartz’s theoretical hypothesis that the more two distant values are in direction in the value circle, the more antagonistic they are. However, this was consistent with the “pluralistic construction” of value hierarchies observed in Chinese college students (Heim et al., 2017). These results indicate that traditional values with strong cultural roots are still alive among the youth in China, despite the growing emphasis on individual goal realization and self-expression (Chen, 2015). Additionally, this pluralistic construction of value hierarchies is easily associated with the dialectical thinking in the self among the Chinese people (Spencer-Rodgers et al., 2004, 2009). Individuals with such thinking styles tend to assume and accept the existence of contradictions (Peng and Nisbett, 1999). Thus, dialectical thinking may make the Chinese youth more receptive to these two values with conflicting basic motivations.

We further identified the neural indicators for the heterogeneous profiles employing resting-state fMRI. In the resting state, the neural activity differences across personal value profiles were observed in the insula rather than in the PFC and ACC, which is not totally compatible with the task state fMRI literature. Specifically, spontaneous activity in the right PI was stronger in Chinese emerging adults with *modernized orientation* than in those with *undifferentiated orientation*. Furthermore, for spontaneous activity in the right PI, a significant positive association was found for self-enhancement and a negative trend toward significance for self-transcendence. These results are consistent with previous studies wherein activation patterns in the PI were revealed to be possible classifiers that distinguish between growth-type core values (e.g., self-transcendence and openness to change) and protection-type core values (e.g., self-enhancement and conservation) (Brosch et al., 2012). Given that the PI, as a zone of convergence for interoceptive information, plays a role in detecting salience (Craig, 2009) and anticipating loss (Canessa et al., 2013), one possible explanation is that stronger attention to self-related needs for individuals with high self-enhancement is associated with high sensitivity to subjective sensations at the neurophysiological level. A positive association between high competitive achievement motivation and right PI grey matter density was observed in a VBM study (Takeuchi et al., 2014) that supports this inference. One may speculate that in the resting state, spontaneous activity in the low-level brain system responsible for visceral sensations may be the neurologically encoded expression of fundamental concerns about self-needs or others’ needs. However, given the relatively small effect size, this analysis should be considered exploratory and needs to be replicated.

The results showed a stronger FC of the MI with the ITG, TG, and POC in Chinese emerging adults with *traditional social orientation* than in those with *modernized orientation* and *undifferentiated orientation*. Furthermore, the FC of the right MI with the right TG was positively correlated with conservation value but negatively correlated with openness to change value. Although the interindividual differences in value hierarchies have seldom been examined, the results are comparable to the results of previous cultural neuroscience research, which reported that compared to Westerners with individualistic self-construal, East Asians with collectivistic self-construal showed larger cortical structures in the temporal regions (Tang et al., 2018) and a higher degree of global efficiency in the temporal regions (Luo et al., 2022). The MI is considered to be a neural marker for bottom-up self-referential processing during the resting state (Frewen et al., 2020). Occipital and temporal lobe activation has been observed in social perception and social cognitive tasks, such as retrieving social knowledge (Olson et al., 2013), making context-based attributions, and inferring others’ mental states (Pehrs et al., 2017). Therefore, this result could be explained by the fact that compared to Chinese emerging adults with *modernized orientation* and *undifferentiated orientation*, those with *traditional social orientation* value the fundamental relatedness of the self to significant others more, and efficient processing of self-related and social-related information at the neural level may facilitate the achievement of this goal.

Conversely, compared to Chinese emerging adults with *modernized orientation* and *undifferentiated orientation*, those with *traditional social orientation* had weaker functional connections within the right MI. This result could be explained by the differences in “ideal affect” (the affective states that people value and would ideally like to feel) resulting from different value hierarchies. Cross-cultural studies have found that, compared to Europeans and Americans valuing high-arousal positive emotions, Chinese people with collectivist values are more likely to appreciate low-arousal positive emotions (Tsai et al., 2006), and they are likely to regulate their emotions by suppressing them (Matsumoto et al., 2008; Ford and Mauss, 2015). de Greck et al. (2012) found that the hemodynamic responses of MI negatively correlated with interdependence scores. Studies have also revealed that the expressive suppression strategies for emotion regulation in non-social contexts are associated with reduced activity in the middle insula (Vrticka et al., 2011). Chinese emerging adults with *traditional social orientation* prefer to adjust their own needs to fit those of their environment and maintain low-arousal emotional states. This preference may be related to the suppression of activity within the MI. Taken together, our results highlight that differences may exist in brain regions for self-relevance and other-relevance processing, subjective sensations, and emotional responses across value hierarchy types.

## Limitations

Some limitations of this study should be addressed in future studies. First, our sample allowed us to identify neural correlates of personal value profiles. However, a relatively high-intelligence sample of college students means that the generalization of the findings obtained from this study remains limited. Future studies with heterogeneous samples (e.g., with greater differences in age and education level) should replicate these findings. Additionally, although the influence of the relatively gender-unbalanced sample on profile creation was negligible in this study, further consideration of the gender differences or replication of our findings in a more gender-balanced sample is recommended. Second, although we centered participants’ responses around their own mean response and converted absolute ratings into relative priorities to overcome differences in response styles on rating methods, conceivably, individuals may overstate or understate their responses based on social expectations, especially in personal value surveys. Future studies should employ multiple approaches (e.g., implicit association approach and free-formatted texts analysis). Finally, the relational nature of these data and the small effect size mean that any assertions regarding causality should be made with caution. Future research using longitudinal assessments and experimental research designs (e.g., assessing neural processing differences of value hierarchy in social/non-social scenarios and perception/emotion/cognitive tasks) are therefore recommended in order to better elucidate causal patterns between value hierarchy and the neural indicators found in this study.

## Conclusion

To summarize, three personal value profiles were identified in Chinese emerging adults, indicating the universality and specificity of individual value transformations during the modernization process. At the neural level, the differences in these personal value profiles are represented in the resting state by spontaneous activity in the right PI involved in self-related information perception, FC of the MI with TG, POC, ITG, and BG involved in self-relevance and other-relevance processing, and FC within the MI involved in emotional responses. The representation of value hierarchies by the insula in the task-free state may imply a preparatory state in which individual values guide cognition, emotion, and behavior in the future.

## Data availability statement

The original contributions presented in the study are included in the article/Supplementary material, further inquiries can be directed to the corresponding authors.

## Ethics statement

The studies involving humans were approved by The Chongqing Ninth People’s Hospital Review Board, China approved this study. The studies were conducted in accordance with the local legislation and institutional requirements. The participants provided their written informed consent to participate in this study.

## Author contributions

J-QX: conceptualization, methodology, formal analysis, investigation, and writing – original draft. YT, JH, and M-ZY: formal analysis and writing - review & editing. Y-DS and Y-JS: investigation. KC: conceptualization, resources, funding acquisition, supervision, and writing – review & editing. GF: conceptualization, methodology, and writing – review & editing. JQ: conceptualization, methodology, investigation, and resources. All authors contributed to the article and approved the submitted version.
