# Supplementary material for: The neural correlates of value hierarchies: a prospective typology based on personal value profiles of emerging adults
Source: Front Psychol. 2023 Dec 18;14:1224911. doi: 10.3389/fpsyg.2023.1224911 (PMC10758175; doi:10.3389/fpsyg.2023.1224911)
Supplement: Supplementary file 1 [file Table_1.DOCX]

**Supporting Information for**

**The Neural Correlates of Value Hierarchies: A Prospective Typology Based on Personal Value Profiles of Emerging Adults**

**Imaging data acquisition and preprocessing**

240 volumes were acquired using a gradient Echo Planar Imaging (EPI) sequence: time repetitive (TR) = 2000 ms, time echo (TE) = 30 ms, slices = 62, field of view (FoV) = 224 mm, base resolution = 112, flip angle (FA) = 90°, dist.factor = 15 %, phase enc.dir = P>>A, accel. factor slice = 2, echo spacing = 0.54 ms. Corresponding field mappings were scanned with following parameters: TR/TE1/TE2 = 620 ms/4.92 ms/7.38 ms, Voxel size = 2.0 × 2.0 × 2.0 mm, slices = 62, FoV = 224 mm, base resolution = 112, dist.factor = 15 %, FA = 60°.

240 volumes were preprocessed by slice-timing, motion-correcting, spatial normalizing, and spatial smoothing. Participants with maximal head motion greater than 2 mm translation or 2.0° rotation were excluded from the study. Field maps were gathered and utilized to produce voxel displacement maps (VDM) to correct spin-echo Echo Planar Imaging (EPI) distortion. Realign & Unwarp were used to apply the FieldMap correction (i.e., the resulting VDM file) to the EPI time series. When Realign & Unwarp do not adequately model dynamic distortions, the FieldMap Toolbox’s ‘Apply VDM’ option (SPM Manual, https://www.fil.ion.ucl.ac.uk/spm/data/fieldmap) can be employed. For spatial normalization, diffeomorphic anatomical registration using exponentiated lie algebra (DARTEL), an image algorithm for the co-registration and spatial normalization of MRI data (Ashburner, 2007), was used. Subsequently, all images were smoothed by convolving them using an isotropic Gaussian kernel of 6 mm full-width at half maximum (FWHM). Nuisance covariates, including the Friston 24 motion parameters (Friston et al., 1996), cerebrospinal fluid, and white matter signals (using a CompCor method, Muschelli et al., 2014) were regressed out. A bandpass filter (0.008–0.09 Hz) was applied. It should be noted that, in the subsequent low-frequency amplitude analysis, we used the data before filtering.

**zALFF differences of ROIs between personal value profiles**

Table 3. The analysis of zALFF differences between personal value profiles

| Labels | Regions | MNI-coordinates | | | ANOVA | | | |
| --- | --- | --- | --- | --- | --- | --- | --- | --- |
|  |  | x(mm) | y(mm) | z(mm) | F(2, 607) | ***p***_un_**_corr_** | ***p*_corr_** | *η^2^_p_* |
| PFC | | | | | | | | |
| Label1 | Right vmPFC | 6 | 64 | 3 | 0.58 | 0.56 | 0.85 | 0.00 |
| Label2 | mPFC | 0 | 51 | 32 | 3.68 | 0.03 | 0.23 | 0.01 |
| Label3 | Left aPFC | -25 | 51 | 27 | 3.31 | 0.04 | 0.23 | 0.01 |
| Label4 | Right vmPFC | 9 | 51 | 16 | 0.94 | 0.39 | 0.85 | 0.00 |
| Label5 | Left vmPFC | -6 | 50 | -1 | 0.44 | 0.64 | 0.87 | 0.00 |
| Label6 | Left vmPFC | -11 | 45 | 17 | 0.08 | 0.92 | 0.95 | 0.00 |
| Label7 | Right vmPFC | 8 | 42 | -5 | 0.59 | 0.56 | 0.85 | 0.00 |
| Label9 | Right vlPFC | 46 | 39 | -15 | 3.31 | 0.04 | 0.23 | 0.01 |
| Label35 | Right aPFC | 29 | 57 | 18 | 0.72 | 0.49 | 0.85 | 0.00 |
| Label36 | Left aPFC | -29 | 57 | 10 | 0.28 | 0.76 | 0.95 | 0.00 |
| Label37 | Right vent aPFC | 42 | 48 | -3 | 1.77 | 0.17 | 0.81 | 0.01 |
| Label38 | Left vent aPFC | -43 | 47 | 2 | 0.54 | 0.58 | 0.85 | 0.00 |
| Label39 | Right vlPFC | 39 | 42 | 16 | 1.01 | 0.37 | 0.85 | 0.00 |
| Label40 | Right dlPFC | 40 | 36 | 29 | 0.58 | 0.56 | 0.85 | 0.00 |
| Label42 | Right dlPFC | 46 | 28 | 31 | 0.09 | 0.91 | 0.95 | 0.00 |
| Label43 | Right vPFC | -52 | 28 | 17 | 1.48 | 0.23 | 0.85 | 0.00 |
| Label44 | Left dlPFC | -44 | 27 | 33 | 0.05 | 0.95 | 0.95 | 0.00 |
| Label56 | Right aPFC | 27 | 49 | 26 | 0.76 | 0.47 | 0.85 | 0.00 |
| Label57 | Right vPFC | 34 | 32 | 7 | 0.20 | 0.82 | 0.95 | 0.00 |
| ACC | | | | | | | | |
| Label8 | Right ACC | 9 | 39 | 20 | 1 | 0.34 | 0.55 | 0.00 |
| Label41 | Left ACC | -1 | 28 | 40 | 1 | 0.34 | 0.55 | 0.00 |
| Label58 | Left ACC | -2 | 30 | 27 | 1 | 0.55 | 0.55 | 0.00 |
| Label61 | Right dACC | 9 | 20 | 34 | 1 | 0.53 | 0.55 | 0.00 |
| Insula | | | | | | | | |
| Label60 | Right AI | 38 | 21 | -1 | 1.56 | 0.21 | 0.78 | 0.01 |
| Label62 | Left AI | -36 | 18 | 2 | 0.45 | 0.64 | 0.78 | 0.00 |
| Label69 | Right MI | 37 | -2 | -3 | 0.93 | 0.39 | 0.78 | 0.00 |
| Label73 | Right MI | 32 | -12 | 2 | 0.44 | 0.64 | 0.78 | 0.00 |
| Label74 | Left MI | -30 | -14 | 1 | 0.32 | 0.72 | 0.78 | 0.00 |
| Label76 | Left PI | -30 | -28 | 9 | 0.58 | 0.56 | 0.78 | 0.00 |
| Label96 | Left MI | -42 | -3 | 11 | 0.97 | 0.38 | 0.78 | 0.00 |
| Label103 | Right MI | 33 | -12 | 16 | 0.85 | 0.43 | 0.78 | 0.00 |
| Label104 | Left MI | -36 | -12 | 15 | 0.25 | 0.78 | 0.78 | 0.00 |
| Label113 | Right PI | 42 | -24 | 17 | 5.42 | 0.00 | 0.04 | 0.02 |

vmPFC = ventromedial prefrontal cortex, mPFC = medial prefrontal cortex, aPFC = anterior prefrontal cortex, vlPFC = ventrolateral prefrontal cortex, dlPFC = dorsolateral prefrontal cortex, vPFC = vent prefrontal cortex, ACC = anterior cingulate cortex, dACC = dorsal anterior cingulate cortex, AI = anterior insula, MI = middle insula, PI = posterior insula.

**External validation results**

Table 4. The evaluation of classification performance

| classification | training features | AUC | Accuracy rate | Specificity rate | Sensitivity rate | F-score |
| --- | --- | --- | --- | --- | --- | --- |
| P1-P2 | zFC of left middle insula with left inferior temporal  zFC of left middle insula with right temporal  zFC within the right middle insula | 0.72^***^ | 0.64^**^ | 65% | 64% | 63% |
| P1-P3 | zFC of left middle insula with left inferior temporal  zFC of right middle insula with right temporal  zFC of right middle insula with right posterior occipital  zFC within the right middle insula | 0.69^***^ | 0.72^**^ | 58% | 64% | 57% |
| P2-P3 | zALFF of right posterior insula  zFC of right middle insula with left basal ganglia | 0.63^***^ | 0.62^*^ | 53% | 56% | 50% |

^*^*p* _permutation tests_ < 0.05, ^**^*p* _permutation tests_ < 0.01, ^***^*p* _permutation tests_ < 0.001. P1 = profile 1 (*traditional social orientation*), P2 = profile 2 (*modernized orientation*), P3 = profile 3 (*multiple value orientation*).

**Reference**

Ashburner, J. (2007). A fast diffeomorphic image registration algorithm. *NeuroImage*, *38*(1), 95–113. <https://doi.org/10.1016/j.neuroimage.2007.07.007>

Friston, K. J., Williams, S., Howard, R., Frackowiak, R. S. J., & Turner, R. (1996). Movement-related effects in fMRI time-series. *Magnetic Resonance in Medicine*, *35*(3), 346–355. https://doi.org/10.1002/mrm.1910350312

Muschelli, J., Nebel, M. B., Caffo, B. S., Barber, A. D., Pekar, J. J., & Mostofsky, S. H. (2014). Reduction of motion-related artifacts in resting state fMRI using aCompCor. *NeuroImage*, *96*, 22–35. https://doi.org/10.1016/j.neuroimage.2014.03.028
